# A Community-enabled Readiness for first 1000 Days Learning Ecosystem (CRADLE) for first-time families: study protocol of a three-arm randomised controlled trial

**DOI:** 10.1186/s13063-021-05144-5

**Published:** 2021-03-06

**Authors:** See Ling Loy, Thilagamangai, Joyce Teo, Sze Wern Chan, Nurul Khairani Abdul Razak, Oh. Moh Chay, Kee Chong Ng

**Affiliations:** 1 Department of Reproductive Medicine, KK Women’s and Children’s Hospital, 100 Bukit Timah Road, Singapore, 229899 Singapore; 2 Duke-NUS Medical School, 8 College Road, Singapore, 169857 Singapore; 3 Singapore Institute for Clinical Sciences, Agency for Science, Technology and Research (A*STAR), 30 Medical Drive, Singapore, 117609 Singapore; 4 Division of Nursing, KK Women’s and Children’s Hospital, Singapore, 100 Bukit Timah Road, Singapore, 229899 Singapore; 5 Medical Board, KK Women’s and Children’s Hospital, 100 Bukit Timah Road, Singapore, 229899 Singapore; 6 Department of Paediatrics, KK Women’s and Children’s Hospital, 100 Bukit Timah Road, Singapore, 229899 Singapore; 7 Yong Loo Lin School of Medicine, National University of Singapore, National University Health System, Singapore, 119228 Singapore; 8 Lee Kong Chian School of Medicine, Nanyang Technological University, 11 Mandalay Road, Singapore, 308232 Singapore

**Keywords:** Choice architecture, First-time parent, Midwife, Nudge, Randomised controlled trial, Self-efficacy

## Abstract

**Background:**

Enhanced parenting self-efficacy (PSE) contributes to positive parenting and future parental-child health. First-time parents, in particular, are in need of support since the pregnancy until post-delivery to strengthen their early PSE. However, there is a lack of effective and sustainable relevant programmes in the community. The Community-enabled Readiness for first 1000 Days Learning Ecosystem (CRADLE) aims to develop a self-learning eco-community throughout the pregnancy and early childhood to promote PSE among first-time parents. We apply choice architecture strategy using behavioural nudges and midwife-led continuity care during the first 1000 days, and test their effects on PSE and mother-child health for first-time families in Singapore.

**Methods:**

This three-arm randomised controlled trial will recruit up to 750 pregnant women from the KK Women’s and Children’s Hospital, Singapore. Participants will be randomly assigned to receive: (1) standard routine care; (2) behavioural nudges (text messages) along with the use of a social media platform; or (3) midwife-led continuity care involving individualised teleconferencing sessions, during pregnancy and post-delivery. Using web-based questionnaires, participants will be assessed for baseline socio-demography and health status in the first visit, with follow-up assessments in the third trimester, at birth, 6-week (primary end-point), 6-, 12-, 18- and 24-month post-delivery. The primary outcome is PSE. Secondary outcomes include health and birth experience, mental wellness, feeding practice, maternal and child nutritional status. Intention-to-treat and per-protocol analyses will be performed using general linear models to test the effects of interventions across three arms. Recruitment has begun in June 2020 and is estimated to complete in September 2022.

**Discussion:**

This study may identify a sustainable effective strategy in the community by helping first-time parents to have a positive experience during the pregnancy, childbirth and parenthood, leading to an enhanced PSE and health outcomes for both mother and child. Findings from this study will provide insight into the implementation of early parenting and mother-child care programmes.

**Trial registration:**

ClinicalTrials.gov NCT04275765. Registered on 19 February 2020.

**Supplementary Information:**

The online version contains supplementary material available at 10.1186/s13063-021-05144-5.

## Background

The “Developmental Origins of Health and Disease (DOHaD)” paradigm underlies the influences of early-life environment on health and disease development over the lifespan [1, 2]. Strong evidence in humans have shown optimal nurturing during pregnancy and after delivery for both mother and child, which are critically important to build an optimal foundation for subsequent long-term maternal-child health [3, 4]. These involve ensuring an optimal psychological and physical health across the pregnancy, lactation and early childhood, as covered by the window of the first 1000 days of life, i.e. 280 days of gestation plus 730 days of the first two postnatal years [5]. The DOHaD or the first 1000 days concept is not just about academic science, but also about education, policy making and everyday practice for individuals [3]. However, to date, most recommendations for pregnant women and young children have yet to take long-term health consequences of early life condition into account [4].

Mental wellness and nutrition, serve as the key components in the psychological and physical health. With the onset of pregnancy, there is a great need to ensure that expectant parents are mentally and physically well prepared to cope with the developing pregnancy, the childbirth and the new life with their babies [6]. On top of building a nurturing environment, this paves the way to promote and enhance early parenting self-efficacy (PSE), as evidenced by the positive link between prenatal mental wellbeing and PSE [7]. PSE, defined as parents’ belief in their ability to perform the parenting role successfully [8], has emerged as an important clinical target for intervention due to its impact on parent and child wellbeing [9]. High PSE is related to effective parenting [10] and great parental competency [11]; low PSE appears to be a salient risk factor of parental depression [12, 13] and is closely linked to long-term harm in child physical and mental development [14–16]. Improving early PSE is thus important towards building a healthy environment for children and for parents, promoting long-term positive health outcomes.

Bandura summarised several factors that could impinge on PSE, including the lack of social support, parental depression, and temperamentally difficult infant and child health problems [17]. This is particularly relevant to first-time families where low PSE is often seen among first-time parents [18]. Specifically, first-time mothers tend to experience difficulties and are vulnerable to emotional distress due to the uncertainties, maladjustments and new challenges faced, including antenatal requirements/demands, intense lifestyle changes, adaptation as a new parent, physical and financial burden, during both pregnancy and postpartum periods [19–21]. The lack of experience, knowledge and skills in antenatal care, childbirth, breastfeeding, infant care and coping strategies have placed them in great needs to acquire social and professional supports [6, 22, 23]. This highlights the importance of having an engaged effective support for these first-time mothers since the onset of pregnancy to promote their self-efficacy in parenting.

In this digital era, though first-time expectant parents could receive much information via the internet, parental education offered by health care professionals remains relevant due to the need of a trustworthy source of information that can be relied on [24]. Delivery of education through midwives during the antenatal and postpartum periods has shown to be useful, making those parents felt supported and prepared for parenting [24–26], with an increased PSE reported [27]. As reviewed, midwife-led continuity care starting from pregnancy until early parenting period has been associated with positive maternal outcomes and parents’ satisfaction [28]. Importantly, the resulted positive maternal outcomes had contributed to reduced health care cost [29]. Unfortunately, in Singapore, there is no midwife-led continuity care service to help first-time parents in gaining positive parenting experience and building PSE. To scale up the service to make it accessible to people, community-based midwifery care is required.

A simple, cost-effective approach which has shown positive effect on parenting is the provision of parental education by health care professionals through digital platforms. A recent web-based psychoeducational intervention over 6 months post-delivery has shown to improve PSE, reduce postnatal depression and enhance social support among first-time mothers in Singapore [30]. A text messaging programme over 1 year for parents of pre-schoolers in the United States was found to be effective in supporting positive parenting practices [31]. The text messages serve as a powerful nudging tool by targeting behavioural barriers to good parenting [32]. Through messages, the complexity of parenting was broken down into small steps that are easy-to-achieve, along with continuous reinforcement and encouragement to help parents change their parenting behaviours and practices [33]. The use of nudges represents a choice architecture strategy which is widely used in public policy making, to alter people’s behaviour and influence decision making [33–35].

### Aims

The main goal of the Community-enabled Readiness for first 1000 Days Learning Ecosystem (CRADLE) study is to promote and sustain healthy behaviour both for the mother and offspring in the first 1000 days and beyond, through a supportive community-led sharing environment for first-time families. To achieve the goal, we developed a virtual interactive and sharing platform, integrated with choice architecture using calibrated nudges, along with midwife-led care service. Our target is to create a self-learning ecosystem by providing knowledge support and guidance, via interactions with health care professionals and peer learning through e-platform. This will in turn create opportunities for the participants to become empowered and eventually develop as community volunteers for maternity care.

**Fig. 1:**
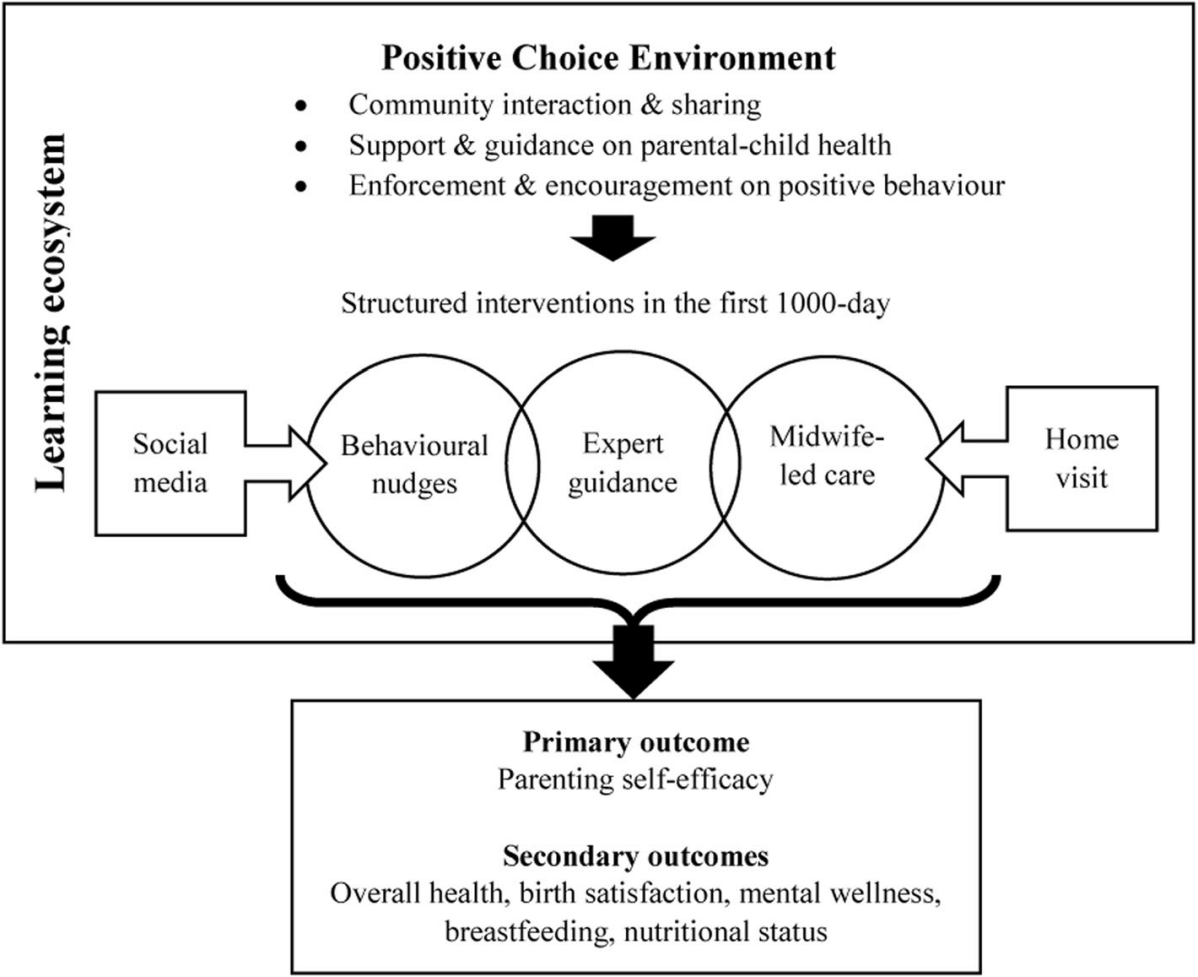
Study framework

Specifically, we aim to examine the effects on PSE and health outcomes in mother and child, with the use of behavioural nudges in the form of text messages (a choice architecture technique) and midwife-led care service, from pregnancy until the first 2 years post-delivery. We hypothesise that during the first 1000 days, the use of virtual nudging tool in the form of text messages supplemented with social media, or physical care from midwives supplemented with individualised teleconferencing sessions will lead to a better maternal PSE, health and birth experience, mental wellness, and nutritional outcomes in both mother-child, compared with those who are receiving standard routine care. The framework of the study is shown in Fig. 1.

## Methods/design

### Trial design

CRADLE is conceptualised as a three-arm, parallel-group, non-blinded randomised control trial (RCT). The allocation ratio of the control group to two intervention groups is 1:1:1. The three arms comprise: (1) standard routine care; (2) behavioural nudges (text messages) along with the use of a social media platform; (3) midwife-led continuity care involving individualised teleconferencing sessions. The protocol has followed the SPIRIT guidelines (see Additional file 1). The trial registration number of the study is ClinicalTrials.gov NCT04275765.

**Fig. 2:**
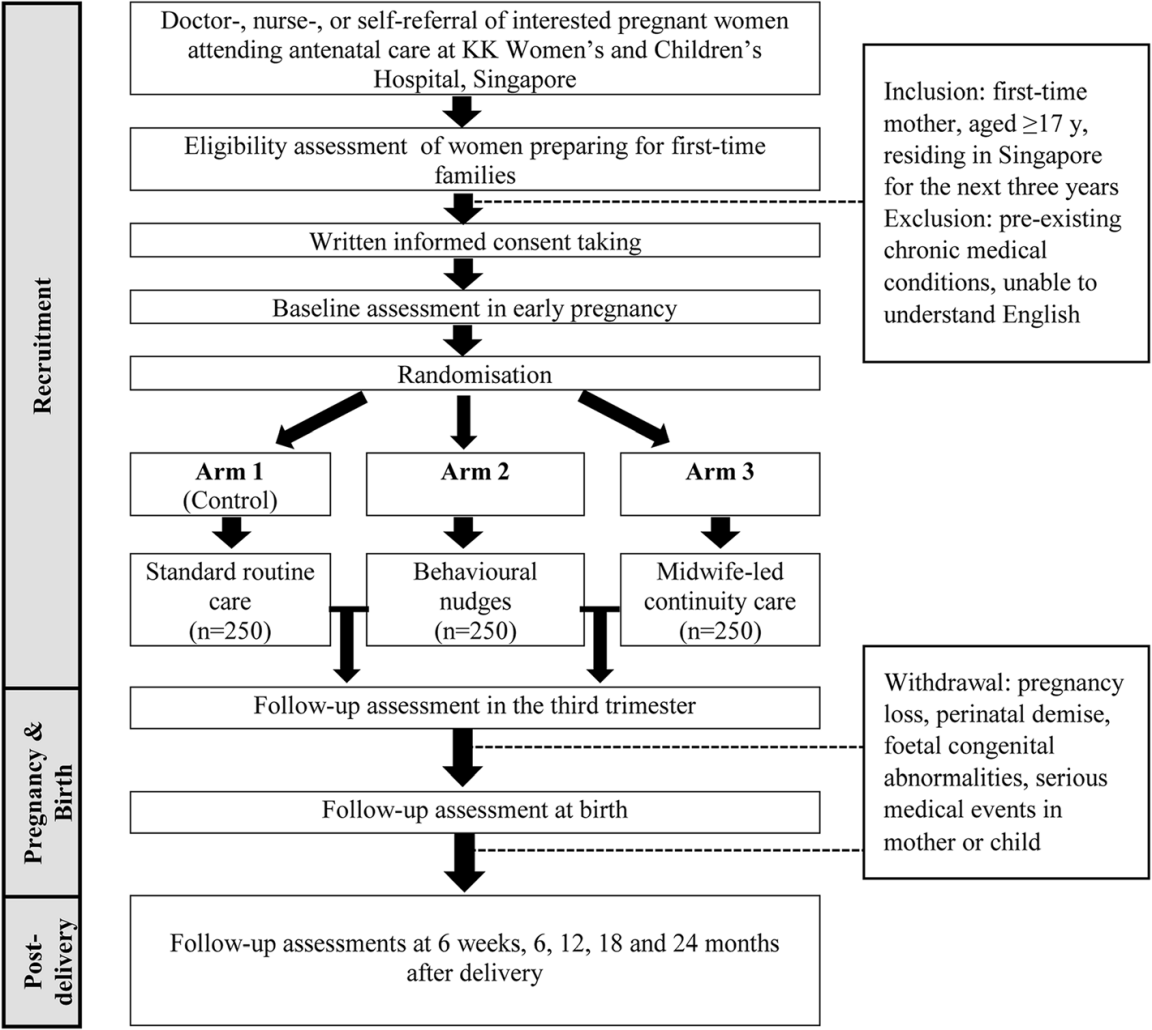
Study flowchart

The flow of the trial is shown in Fig. 2. Following informed consent at the recruitment visit, a baseline assessment on maternal sociodemographic characteristics and health status will be conducted, followed by randomisation to the control or intervention group (Arm 2 or 3) using the electronic randomisation list. Depending on the follow-up time-point (i.e. 30-week, at birth, 6-week, 6-, 12-, 18-, and 24-month post-delivery), every participant is required to complete few sets of online questionnaires for assessments on PSE, health and birth experience, mental wellness and feeding practice. A 2-week timeframe is given to the participants to complete the questionnaires, depending on their preferable schedule and availability within the period. Maternal and child anthropometric measures including weight and height are also assessed. Details of study measures and questionnaires used according to the visit time points are presented in Table 1.

**Table 1:** Data collection and study instruments

	Baseline visit	Third trimester	Birth	6 weeks	6 months	12 months	18 months	24 months
**Socio-demography**								
Age	√							
Ethnicity	√							
Family structure	√							
Education	√							
**Health and birth experience**								
PROMIS Global-10 scale^*^	√	√			√		√	
Birth Satisfaction Scale-Revised^*^			√					
**Mental wellness**								
Patient Health Questionaire-2^*^	√	√		√	√			
Edinburgh Postnatal Depression Scale	√	√		√	√			
**Nutrition**								
Breastfeeding success			√	√	√			
Breastfeeding confidence			√					
Breastfeeding Self-Efficacy Score-Short Form^*^			√	√	√			
Infant Feeding Questionnaire							√	
Maternal and child weight/height	√	√	√	√	√	√	√	√
**Parenting self-efficacy**								
TOPSE^*^				√	√	√	√	√
Parental Sense of Competence Scale^*^				√	√	√	√	√
**Medical records retrieval**								
Obstetric and delivery outcomes			√					

*From the International Consortium for Health Outcomes Measurement (ICHOM) Standard Set of Outcome Measures for Pregnancy and Childbirth

*PROMIS* Patient-Reported Outcomes Measurement Information System, *TOPSE* Tool to measure Parenting Self-Efficacy

### Recruitment

Recruitment is conducted at the KK Women’s and Children’s Hospital (KKH), Singapore. In this study, we target to recruit up to 750 pregnant women visiting antenatal clinics of KKH during their early pregnancy. Recruitment brochures, posters and banners that contain general information and selection criteria of the study are placed at different locations, such as hospital lifts and clinics. Interested women self-contact the study team by email or phone, or to be referred by doctors or nurses.

Inclusion criteria are women who meet the following: first-time pregnant mother, aged 17 years and above, going to reside in Singapore for the next 3 years, able to understand English (or with a family member who is able to assist), and to provide written, informed consent. Exclusion criteria are women with medical conditions such as serious chronic diseases and high risk complicated pregnancies. Throughout the study period, participants who experience loss of pregnancy, perinatal demise, congenital abnormalities in offspring, serious medical events in mother/child, unable to comply with study protocol or wish to discontinue their participation will be withdrawn from the study. For participants who withdraw from the study, consent will be sought to retain and analyse their data until the withdrawal time-point.

### The intervention

Arm 2 and Arm 3 interventions are designed based on the framework that can eventually lead to a self-learning ecosystem in the community to improve early PSE and health outcomes for first-time families.

In Arm 2, we interact with the participants virtually. The participants in this intervention arm will receive weekly mobile messages on important information related to antenatal care, breastfeeding, infant care, child growth and development, and postpartum care. These messages are targeted and time specific according to stages in the pregnancy and early childhood. There were designed by the study team’s health experts as reference to the Ready4K text messaging programme [31], serving as nudges to influence behaviour and guide first-time families in their pregnancy and early parenting journey. In order to create an interactive and sharing environment with peer support, participants are enrolled automatically to a private social media platform group, where they are encouraged to share information, post photos and interact with each other. More detailed and updated health information related to maternity care and parenting skills are available in the social media platform to provide guidance and support to the participants, as well as to encourage self-learning. Meanwhile, participants can post any query on health concern in their first 1000 days, which will be addressed by the study team’s experts. This is a restricted and by-invitation-only social media forum. The contents are monitored by the research staff to ensure this sharing platform contains trustworthy information. We envision that motivated participants will be empowered and eventually, emerge as chat group leaders in this platform.

In Arm 3, we conduct midwife-led continuity care involving individual teleconferencing sessions. Participants will be encountered by midwives who interact with them at various time points throughout the study. During pregnancy, one session that lasts for 15–20 min is held in the antenatal clinic per trimester. Any questions pertaining to pregnancy or childbirth can be clarified with the midwife. During post-delivery, the same midwife will conduct individualised teleconferencing sessions at 1- and 2-week postpartum, and call to check for progression at 6-week, 3- and 6-month postpartum. Participants can discuss any concern relating to postpartum health, infant care, breastfeeding, child growth and development with the midwife. In the case when there is uncertainty about queries asked by the participant, advice from the study team’s health experts will be sought. We envision that motivated participants will be empowered and eventually, emerge as support volunteers assisting in midwifery care delivery in the community.

### Outcome measures

We quantify the impact of interventions by measuring PSE in first-time mothers, along with specific health outcomes.

#### Primary outcome

The primary outcome of this RCT is PSE, measured using the Tool to measure Parenting Self-Efficacy (TOPSE) and the Parental Sense of Competence Scale (PSOC) at 6-week as the primary endpoint, followed by 6-, 12-, 18- and 24-month post-delivery. We include both TOPSE and PSOC, as the combination use of these two tools provides a comprehensive measure on the domain-specific and domain-general self-efficacy components. In addition, we are able to evaluate, following suggestions by Bandura [36], any differences in employing two assessment strategies operating at different levels of specificity [8].

The TOPSE is a multidimensional, self-report measure designed to assess parents’ perceived ability to parent their children, which took into account the views and experience of parents from a range of cultural and social backgrounds [37, 38]. The theoretical underpinning of TOPSE is based on self-efficacy theory developed by Bandura [36, 39]. It consists of 48 items which are divided into eight subsections: (1) emotion and affection, (2) play and enjoyment, (3) empathy and understanding, (4) control, (5) discipline and setting boundaries, (6) pressures, (7) self-acceptance and (8) learning and knowledge. Participants will be requested to rate items using a 11-point Likert scale ranging from 0 (completely disagree) to 10 (completely agree). The higher the score, the higher the level of PSE.

The PSOC is a self-report instrument designed to measure parenting satisfaction and efficacy [40]. It is a domain-general measure with items describing common parental ideas regardless of the child’s age and the specific tasks that the parent has to face [40]. Although the PSOC has mainly been used in parents with children, it has been found to be valid for use in parents with infants [41, 42]. It consists of 17 items scored on a 6-point Likert scale ranging from 1 (strongly disagree) to 6 (strongly agree). The higher the score, the higher the level of parenting satisfaction and self-efficacy.

#### Secondary outcomes

Secondary outcomes include health and birth experience, mental wellness and nutritional outcomes, assessed using validated patient reported outcome measurements, including the use of the International Consortium for Health Outcomes Measurement (ICHOM) Standard Set of Outcome Measures for Pregnancy and Childbirth [43] and by anthropometric measures. Time points of assessment and ICHOM tools are indicated in Table 1.

Health experience will be assessed using the Patient Reported Outcomes Measurement Information System (PROMIS) Global-10 [44]. The instrument consists of 10 items representing general health, physical health, mental health, social health, pain, fatigue, and overall perceived quality of life. Nine of the 10 items are scored on a 5-point Likert scale ranging from 1 (poor/not at all) to 5 (excellent/completely). The pain – 10th item, is scored from 0 (no pain) to 10 (worst imaginable pain). This instrument has been found valid to be used for maternity population [45].

Birth experience will be assessed using the Birth Satisfaction Scale-Revised [46]. The instrument measures women’s perceptions on quality of care provision, women’s personal attributes and stress experienced during labour. It consists of 10 items rated on a 5-point Likert scale ranging from 0 (strongly disagree) to 4 (strongly agree).

Mental wellness will be assessed using the Patient Health Questionnaire-2 (PHQ-2) and the Edinburg Postnatal Depression Scale (EPDS). The PHQ-2 consists of 2 items rated on a 4-point Likert scale from 0 (not all all) to 3 (nearly every day), to screen for depression as a first step approach [47]. EPDS will be used when a woman scores a three or above on the PHQ-2, in order to further assess depressive symptoms in details. Although EPDS was originally designed to measure postnatal depression [48], it has been shown to be valid in assessing antenatal depression [49]. It consists of 10 items rated on a 4-point Likert scale, ranging from 0 (as much as I always could) to 3 (not at all).

Nutritional outcomes are assessed based on breastfeeding and infant feeding practices, as well as anthropometric measures. We evaluate breastfeeding in terms of breastfeeding success, breastfeeding confidence and breastfeeding self-efficacy. Breastfeeding success is measured according to feeding mode in the past 7 days, with options of ‘only breast milk’, ‘combination of breast milk, formula and/or water’, and ‘only formula, water or other liquids but not breast milk’ [50]. Breastfeeding confidence is evaluated based on a 5-point Likert scale from 1 (not at all) to 5 (very confident) [50]. Breastfeeding Self-Efficacy Score-Short Form is used to assess maternal confidence in her ability to breastfeed her new baby [51]. The instrument consists of 14 items rated on a 5-point Likert scale, ranging from 1 (not at all) to 5 (very confident). The Infant Feeding Questionnaire (IFQ) is used to assess maternal feeding beliefs and practices [52]. This tool has been shown to be valid for use in our population [53]. IFQ is a 28-item questionnaire rated on a 5-point Likert scale, ranging from 1 (never/disagree a lot) to 5 (always/agree a lot). Nutritional status for both mother and child is assessed throughout the study period, by evaluating maternal weight status across pregnancy and during postpartum, and child growth over the first 2 years, i.e. weight and length.

### Sample size

Based on a previous parenting programme in childhood, a moderate effect (*d* = 0.4) on PSE was reported [54]. We postulate that with our early interventions, a difference of *d* = 0.35 in the overall TOPSE (primary outcome) score between intervention and control arms will be detected. In order to achieve a power of at least 80% with a two-sided significance level at 5%, a total of 150 participants are required for each arm. Assuming a dropout rate of 66% during the first 1000-day follow-up, the necessary sample size is 250 participants per arm, resulting in a total of 750 participants for three arms.

### Statistical analysis

One-way analysis of variance (ANOVA) and chi-square tests or Fisher’s exact tests will be used to compare differences in baseline characteristics among the three arms. The primary analysis will be according to the intention-to-treat principle, i.e. all participants who were assigned to a condition. Differences in outcomes across three arms at each time-point will be compared using the one-way analysis of covariance (ANCOVA) (General Linear Model, GLM), adjusting for baseline characteristics and measures. Bonferroni correction will be performed for pair-wise comparisons. Linear mixed models will be used to compare the outcomes across time points among the three arms, adjusting for baseline characteristics and measures. Additionally, a priori sensitivity analysis will be conducted for complete cases, i.e. participants with complete follow-up and data. There are no formal planned interim analyses of the primary outcome, but progress reports on all data issues will be presented to investigators of the study team. The level of statistical significance will be set at 0.05 (two-tailed) and 95% confidence intervals will be calculated.

### Quality control

Research staff who are responsible to recruit and follow-up participants received training on recruitment approach, informed consent taking, randomisation procedure, online questionnaires management, mobile messages (nudges) and reminders deliveries. Midwives received special training on midwifery continuous care service. Monthly meetings will be held with investigators and/or project manager to review study procedures, recruitment strategy, participants’ feedback and collected data. An annual report on study progress will be prepared. The study will be audited by an independent party annually.

### Data monitoring

All data will be anonymised. Participants’ identifiers will be kept separately in a password-protected file. Electronic data will be managed using an online data capture tool. We will set up a data monitoring team to perform data checking on the completeness, errors and outliers. The staff will perform data checking weekly to ensure questionnaires are submitted by the participants within the 2-week timeframe. To facilitate the data monitoring procedure, a reminder message to check on the data completeness will be triggered automatically by the system after the initial receipt of online questionnaires. If the participants do not submit the questionnaires or with any incomplete section/missing data, research staff will contact the participants to remind or check on the problems faced. A meeting will be held with investigators quarterly to review the data with issues. Paper documents will be kept in a locked cabinet and electronic data will be stored on password-protected computers or hard-disk drives which can only be accessed by research team members. All records will be maintained for at least 6 years after completing the study.

### Patient and public involvement

The research questions, study design, exposure and outcome measures were determined based on a discussion with health experts (clinicians, nurses and researchers) in maternal and child care. Although participants did not directly contribute to the development of research questions and the study design, their needs, preferences and participation burden were considered throughout the process. The results of the study will be disseminated to participants at their request, after the study is completed.

### Ethics and dissemination

Participants will sign a written informed consent after full disclosure and explanation of the study purpose and procedure. In the event of any new information or change in the study protocol that may be relevant to their willingness to continue the study, research staff will contact the participants in timely manner to seek for further consents if required. Participants will be informed that the use of nudges, social media and community midwife care are not yet proven to be a standard care for first-time parents from pregnancy to 2 years post-delivery. The use of these approaches is solely for research purpose. We expect no adverse health effect from the interventions.

The main findings of this study will be published in peer-reviewed international journals. Presentations of the study results will also be carried out in symposia, seminars and conferences. If the interventions are found to produce a positive impact, we will consider a media release to publicise the research findings.

## Discussion

This protocol describes the rationale and design of a three-arm RCT that aims to improve PSE and health outcomes of mother-child for first-time families in Singapore, through a choice architecture approach using behavioural nudges and midwife-led care continuity services during the first 1000 days. The interventions are supported by social media platform, individualised teleconferencing sessions and health care expert guidance to create an interactive, sharing and self-learning environment in the community. The study framework is designed by tapping into existing scientific and medical evidence to guide first-time parents to shape their health behaviour since the onset of pregnancy and to improve self-efficacy in early parenting journey. It translates DOHaD science into advanced disease prevention by intervening at critical windows—the first 1000 days, for optimal promotion of healthy childhood development, and to reduce mental disorders or metabolic diseases in the mother-offspring.

CRADLE is an implementation research, which will contribute to build an evidence-based parenting support system in the community for new parents and new families. Through this study, we will have a better understanding on the needs and ways to well prepare our families for parenting in the first 1000 days, which will impact our population health in long run. Findings from the study will also help us to increase knowledge and experience about effective strategies to promote mental wellbeing and optimal nutrition for pregnant women, postpartum women and young children. This contributes to an effort in breaking intergenerational vicious cycle of chronic disease, which is a current worldwide issue [55]. Besides, the study has the potential to provide insights to develop a sustainable model of health guidance for first-time families in early parenthood, algorithms in mother-child care, and personalised care paths for distinct subsets of first-time families with specific individualised needs in the first 1000 days.

The greatest strength of this study is the use of an integrated choice architecture approach to test out its effect on PSE using a RCT design through the first 1000 days. Studies in health and education provide evidence that text messaging as nudging tool can be an effective way to change complex, continuous and long-term behaviours, underscoring its potential promote parental involvement [33, 35]. The focus on early life intervention starting since early pregnancy enables us to examine the influences of intervention which is expected to be more effective compared to other RCTs on parenting care that only initiated at post-delivery [30, 56]. Assessments of outcome measures at multiple time-point allow us to monitor and track the health progress longitudinally. Based on the findings, subsequent plans will be considered to examine the feasibility of utilising ICHOM tools and implementing them nationwide to monitor public health in Singapore. We are therefore embarking on a proof-of-concept and proof-of-value study, which is important to address challenges of the know-do gap in real-world setting.

Few limitations are acknowledged. First, the study is restricted by its external validity as all participants will be recruited from one hospital in Singapore. Thus, caution is required to extrapolate the findings to the general population. Nevertheless, KKH houses the largest public maternity unit in Singapore, and manages ~ 30% of all live births in Singapore, across a wide sociodemographic spectrum. We will check for generalisability of findings by comparing differences in basic demographic data between this study and other studies involving larger population of pregnant women in Singapore [57]. Second, the study will only involve participants who are able to understand English, which could limit the generalisability of results to other individuals/families who are non-English speaking. However, English is the first language in Singapore which is widely used and understandable across nations, especially the young who are our primarily samples. Third, there may be a risk of cross-contamination between study arms due to the single hospital setting in the recruitment though different antenatal clinics will be involved. In order to reduce the contamination risk, participants will be instructed not to share their information received with others, except partner and members within the social media platform (Arm 2). Finally, we will target only for mothers but not fathers for the measurement outcomes in this study. This is to reduce variations attributable to differential parental responses arise from differences in paternal and maternal expectations. However, the intervention’s content and context were designed to be applicable for first-time families, i.e. both parents.

## Trial status

Recruitment for the trial has begun on 15 June 2020 and is estimated to complete on 30 September 2022. The current protocol is version 3, 7 July 2020.

## Supplementary Information

**Additional file 1.** SPIRIT 2013 Checklist. Recommended items to address in a clinical trial protocol and related documents.

## Data Availability

This manuscript does not contain any data. Datasets during and/or analysed during the study will be available from the principal investigator (KCN) on reasonable request. The results of the study will be submitted for publication in peer-reviewed journals as soon as possible after analysis.

## Abbreviations

CRADLE: Community-enabled Readiness for 1000 Days Learning Ecosystem
DOHaD: Developmental Origins of Health and Disease
EPDS: Edinburg Postnatal Depression Scale
ICHOM: International Consortium for Health Outcomes Measurement
KKH: KK Women’s and Children’s Hospital
PHQ-2: Patient Health Questionnaire-2
PSE: Parenting self-efficacy
PSOC: Parental Sense of Competence Scale
TOPSE: Tool to measure Parenting Self-Efficacy
RCT: Randomised controlled trial
